# Updated Review of Metal Nanoparticles Fabricated by Green Chemistry Using Natural Extracts: Biosynthesis, Mechanisms, and Applications

**DOI:** 10.3390/bioengineering11111095

**Published:** 2024-10-30

**Authors:** Hesham R. El-Seedi, Mohamed S. Omara, Abdulrahman H. Omar, Mahmoud M. Elakshar, Yousef M. Shoukhba, Hatice Duman, Sercan Karav, Ahmed K. Rashwan, Awg H. El-Seedi, Hamud A. Altaleb, Haiyan Gao, Aamer Saeed, Ohoud A. Jefri, Zhiming Guo, Shaden A. M. Khalifa

**Affiliations:** 1 International Research Center for Food Nutrition and Safety, Jiangsu University, Zhenjiang 212013, China; 2 Department of Chemistry, Faculty of Science, Islamic University of Madinah, Madinah 42351, Saudi Arabia; 3 Department of Chemistry, Faculty of Science, Menoufia University, Shebin El-Kom 32111, Egypt; 4 Botany and Microbiology Department, Faculty of Science, Menoufia University, Menoufia 32111, Egypt; mohamedomarav97@science.menofia.edu.eg (M.S.O.); abdulrahmann0marr@gmail.com (A.H.O.); elakshar.mahmoud789@gmail.com (M.M.E.); youssefmahmoud0106@gmail.com (Y.M.S.); 5 Department of Molecular Biology and Genetics, Çanakkale Onsekiz Mart University, Çanakkale 17000, Turkey; hatice.duman@comu.edu.tr (H.D.); sercankarav@comu.edu.tr (S.K.); 6 Department of Food Science and Nutrition, College of Biosystems Engineering and Food Science, Zhejiang University, Hangzhou 310058, China; a.rashwan@agr.svu.edu.eg; 7 International IT College of Sweden, Stockholm, Hälsobrunnsgatan 6, Arena Academy, 11361 Stockholm, Sweden; awg.elseedi@gmail.com; 8 Zhejiang Key Laboratory of Intelligent Food Logistic and Processing, Key Laboratory of Post-Harvest Handling of Fruits, Ministry of Agriculture and Rural Affairs, Food Science Institute, Zhejiang Academy of Agricultural Sciences, Hangzhou 310021, China; spsghy@163.com; 9 Department of Chemistry, Quaid-I-Azam University, Islamabad 45320, Pakistan; asaeed@qau.edu.pk; 10 Department of Biological Science, Faculty of Science, King Abdulaziz University, Jeddah 21589, Saudi Arabia; jeefrio0@gmail.com; 11 Department of Biology, College of Science, Taibah University, Al-Madinah Al Munawarah 42353, Saudi Arabia; 12 School of Food and Biological Engineering, Jiangsu University, Zhenjiang 212013, China; guozhiming@ujs.edu.cn; 13 Neurology and Psychiatry Department, Capio Saint Göran’s Hospital, Sankt Göransplan 1, 11219 Stockholm, Sweden

**Keywords:** nanoparticles, metallic nanoparticles, green chemistry, biosynthesis, encapsulation

## Abstract

Metallic nanoparticles have found wide applications due to their unique physical and chemical properties. Green biosynthesis using plants, microbes, and plant/microbial extracts provides an environmentally friendly approach for nanoparticle synthesis. This review discusses the mechanisms and factors governing the biosynthesis of metallic nanoparticles such as silver, gold, and zinc using various plant extracts and microorganisms, including bacteria, fungi, and algae. The phytochemicals and biomolecules responsible for reducing metal ions and stabilizing nanoparticles are discussed. Key process parameters like pH, temperature, and precursor concentration affecting particle size are highlighted. Characterization techniques for confirming the formation and properties of nanoparticles are also mentioned. Applications of biosynthesized nanoparticles in areas such as antibacterial delivery, cancer therapy, biosensors, and environmental remediation are reviewed. Challenges in scaling up production and regulating nanoparticle properties are addressed. Power Point 365 was used for creating graphics. Overall, green biosynthesis is an emerging field with opportunities for developing eco-friendly nanomanufacturing platforms using abundant natural resources. Further work on optimizing conditions, standardizing protocols, and exploring new biosources is needed to realize the full potential of this approach.

## 1. Introduction

Nanotechnology has served a prominent promise for the human health and welfare system within a short time. The development in this area initiated the revolution of engineering, biomedical, sensing, and catalytic implications. Nanotechnology is now being applied in diverse fields, including drug/gene delivery, bioimaging, biomimetic materials, diagnostic assays, implant coatings, environmental remediation, renewable energy, and many more [1,2,3,4].

The ability to control nanomaterials at the atomic or molecular scale endows them with novel physicochemical properties distinct from their bulk counterparts. This has enabled the development of advanced nanomaterials and devices with superior performance compared to conventional macro-scale materials [5]. Overall, nanotechnology has opened new frontiers for innovation across industries by harnessing nanoscale effects. Metallic nanoparticles have gained tremendous importance in recent years, exhibiting unique optical, electronic, and catalytic properties [6,7,8]. Conventional methods for metal nanoparticle synthesis often rely on the use of toxic chemicals, which raise environmental and biological safety concerns. As a result, there is growing interest in developing green biosynthesis methods using plant extracts and microorganisms.

Plants and microbes have evolved sophisticated mechanisms to sequester and detoxify metal ions from the environment [9]. They secrete large biomolecules like enzymes, proteins, and phytochemicals, which can effectively reduce metal ions into their respective elemental forms as nanoparticles during the biosynthesis process [10,11]. Compared to chemical and physical approaches, green biosynthesis is energy-efficient, cost-effective, and easily scalable for large-scale production. Importantly, biological synthesis produces nanomaterials with minimal environmental pollution in an eco-friendly manner [12].

Biosynthesis of silver, gold, zinc, selenium, and other metal nanoparticles using plant extracts from fruits, vegetables, herbs, and microbial culture supernatants has been widely reported. However, the exact mechanisms governing the extracellular and intracellular biosynthesis pathways are still not fully understood. Various factors influencing nanoparticle formation, such as metal ion concentration, pH, temperature, and reaction time, also need further investigation. This review aims to provide a comprehensive overview of green biosynthesis of different metal nanoparticles mediated by plants and microorganisms. The aim is to discuss the proposed mechanisms of nanosynthesis and characterize the biomolecule–metal interactions. Finally, the diverse applications of green synthesized nanoparticles in areas such as electronics, photonics, catalysis, sensing, and biomedical science will also be highlighted. Additional studies have been conducted as an extension of our highly cited paper [13] and a part of our ongoing project on metal nanoparticles fabricated by green chemistry using natural extracts [14,15,16].

## 2. Plant-Mediated Synthesis of Metallic Nanoparticles

Plants have shown promise for the sustainable biosynthesis of metallic nanoparticles owing to their phytochemical profile that serves as both reducing and stabilizing agents [17]. Upon exposure of metal salts to plant extracts rich in polyphenols, terpenoids, alkaloids, and other bioactive compounds, metal ions become reduced to form their corresponding nanoparticles in aqueous solution [18]. Where these bioactive compounds act as both reducing and stabilizing agents [19]. Phytochemicals in plant extracts act as redox mediators, donating electrons to metal ions [20]. This drives the conversion of ions to neutral atomic form within the nanometer scale, which is the size of an atom.

The process of metallic nanoparticle synthesis using plant extracts involves the reduction in metal ions by phytochemicals present in the extracts. Phytochemicals like polysaccharides, vitamins, amino acids, proteins, saponins, alkaloids, terpenes, and phenolics play a role in this synthesis. The general steps of green biosynthesis of metal nanoparticles involve mixing a plant or microbial extract containing reducing agents with a metal salt solution. This leads to the reduction of metal ions to atoms and nucleation to form initial nanoparticles.

**Figure 1:**
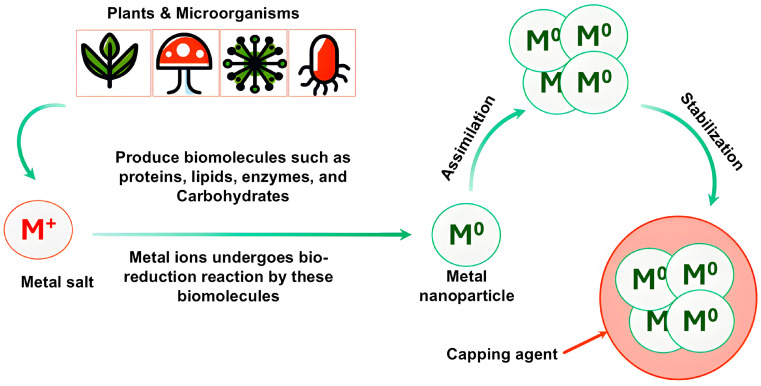
The process of biosynthesis of metal nanoparticles by plants and microorganisms. Plants and microorganisms produce biomolecules that convert metal ions into metallic nanoparticles which the assimilated together and finally stabilized by capping agents.

The growing nanoparticles are then capped and stabilized through electrostatic interactions or steric hindrance provided by biomolecules in the extract (Figure 1). This keeps the fabricated metallic nanoparticles at the nanoscale and prevents unwanted aggregation [21,22].

The specific mechanism of metallic nanoparticle synthesis by plants can vary based on factors like the type of extract, metal salt, reaction conditions, and the presence of enzymes or antioxidants in the extract that influence the reduction process and nanoparticle properties [17,23,24]. However, generally, when metallic salts are added to plant extracts, the biomolecules interact with metal ions via their functional groups like hydroxyl and carboxyl [25]. Two mechanisms underlie this process, i.e., the redox mechanism and the ligand substitution mechanism. In the first mechanism, polyphenols can undergo oxidation–reduction reactions. They become oxidized and donate electrons to metal ions, reducing them from their ionic state to neutral atoms. While during the ligand substitution mechanism, biomolecules directly bind to metal ions via their functional groups, forming metal–biomolecule complexes. Electrons are then transferred from the ligands to the metal ions during this coordination, reducing them to neutral atoms [18,26]. Both mechanisms involve electron donation from phytochemicals. The key difference lies in whether direct complexation or indirect oxidation–reduction drives reduction of metal cations to atoms during green synthesis of nanoparticles.

Table 1 provides a compilation of research findings from over 90 plant species investigated for their ability to synthesize nanoparticles of various metals from the period 2019 to 2023. The plant parts ranged from leaves, stems, roots, barks, fruits, and flowers. However, leaves have emerged as the predominant plant tissue employed for the biosynthesis of metal nanoparticles. They provide a plethora of reduced secondary metabolites due to their primary role in photosynthesis. These metabolites, such as phenolics, flavonoids, terpenoids, alkaloids, and saponins, can act as reducing and capping agents for the formation and stabilization of metal nanoparticles [20]. The ease of collection without harming plants ensures sustainability. Fast regeneration maintains a continual biomass supply, enabling scalability. Therefore, the simplicity of using leaves, along with their natural richness in bioactive compounds, makes them the predominant choice for driving the green synthesis of various metal nanoparticles.

The same plant species can be harnessed for the synthesis of several metallic nanoparticles; for example, the leaf extract of *Abutilon indicum* was used for the synthesis of manganese oxide (MnO) and chromium oxide nanoparticles (CrO NPs) [27,28]. The peel extract and seed extract of *Moringa oleifera* were used for the synthesis of gadolinium oxide (GdO) and silver nanoparticles (AgNPs), respectively [29,30].

**Table 1:** Some plants involved in metallic NP synthesis.

Plant Species	Used Part	NPs	NP Size (nm)	UV Absorption (nm)	Activity	References
*Abutilon indicum*	Leaves	MnO	80 ± 0.5	380 and 460	Antibacterial and anticancer	[27]
*Abutilon indicum*	Leaves	CrO	17–42	280 and 415	Antibacterial, anticancer, and antioxidant	[28]
*Achillea millefolium*	Leaves	Ag	14–18	400–700	Antibacterial and antioxidant	[31]
*Achillea wilhelmsii*	Leaves	Au	2.7–38.7	540	Antibacterial, antioxidant and electrocatalytic activity	[32]
*Aerva tomentosa*	Root	Ag	-	443	Antibacterial and antioxidant	[33]
*Aesculus hippocastanum*	Leaves	Ag	50 ± 5	420–470	Antibacterial and antioxidant	[34]
*Ajuga bractosa*	-	Ag	400	-	Antibacterial	[35]
*Albizia lebbeck*	Stem bark	ZnO	66.25, 82.52, and 112.87	300–800	Antimicrobial and antioxidant	[36]
*Alcornea laxiflora*	Leaves	Ag	20–52	424–435	Antibacterial, photocatalytic degradation, and tyrosinase inhibition	[37]
*Allium ampeloprasum*	Aerial parts	Ag	80–50	300–800	Antioxidant and antibacterial	[38]
*Atalantia monophyla*	Leaves	Ag	8.3	396	Antimicrobial and antioxidant	[39]
*Atropa acuminata*	Leaves	Ag	5–20	428	Antioxidant, anti-inflammatory, anticancer, and larvicidal activities	[40]
*Azadirachta indicia*	-	ZnO	19.57 ± 1.5	-	Antioxidant, antibacterial, and enzyme inhibitor	[41]
*Bauhinia purpurea*	Leaves	Ag and Au	*-*	430 and 560	Anticancer, antioxidant, antimicrobial, and catalytic agents	[42]
*Bidens Pilosa*	Leaf, stem, and root	Ag	17	-	Antimicrobial and anticancer	[43]
*Brassica oleracae*	Leaves	SnO_2_ NPs	3.62–6.34	-	Dye degradation activity	[44]
*Brassica pekinensis*	Leaves	Au	-		Antioxidant and antimicrobial	[45]
*Callistemon viminalis*	Bark	Ag	55	400–435	Antioxidant, antibacterial, and catalytic	[46]
*Cannabis sativa*	Leaves	Ag	69	435	Antibacterial	[47]
*Cayratia pedate*	Leaves	ZnO	52.24	320	Enzyme immobilization	[17]
*Chlorophytum borivilianum*	Leaves	Ag	52	450	Antimicrobial	[48]
*Chromolaena odorata*	Leaves	Ag	27.82–32.89	435	Antibacterial	[49]
*Cinnamomum tamala*	Leaves	FeO	26–35	300–800	Wastewater treatment	[50]
*Cinnamomum zelanicum*	Leaves	Cu	19.55–69.70	-	Antioxidant and anticancer	[51]
*Citrullus colocynthis*	Leaves	ZnO	50–60	374	Anticancer	[52]
*Clinacanthus nutans*	Leaves and stem	Ag	-	600	Antioxidant and antimicrobial	[53]
*Coptis chinensis*	Leaves	Ag	6–45	450	Antibacterial and anticancer	[54]
*Coriandrum sativum*	Leaves	Au	32.96 ± 5.2	540–550	Antioxidant and analgesic activity	[55]
*Costus igneus*	Leaves	ZnO	26.55	365	Antidiabetic, antioxidant, antibacterial, and antibiofilm	[56]
*Curcuma wenyujin*	Herbal	Au	100 nm	530	Anticancer	[57]
* Derris trifoliata *	Seeds	Ag	16.05 ± 5.0	360	Antioxidant, antibacterial, and antiproliferative activity	[58]
*Dodonaea viscosa*	Leaves	Ag	20–100	441–564	Antibacterial and anticancer	[59]
*Emblica Phyllanthus*	Leaves	Ag	30–65	425	Antidiabetic and hypolipidemic	[60]
*Eryngium planum*	Leaves	Ag/FeO	60	450	Noncorrosive heterogeneous catalysts	[61]
*Euphorbia tirucalli*	Arial parts	Mg andCoO	100 nm–1 µm	305 and 508	Antiproliferative agents for cancer	[62]
*Fagonia cretica*	-	Ag	11–15	440	Antimicrobial	[63]
*Ganoderma lucidum*	Fruit bodies	Ag	10.72	409	Anticancer	[64]
*Garcinia Kola*	Leaves	Ag	28.8	425.18	Antibacterial	[65]
*Gelidium pusillum*	-	Au	7–17	529	Anticancer	[66]
*Gracilaria crassa*	Leaves	Au	32.0 nm ± 4.0 nm (mean ± S EM)	-	Ecotoxicological potential	[67]
*Hibiscus cannabinus*	Leaves	Ag	9	446	Antimicrobial	[68]
*Hylotelephium telephium*	Flowers	CuO and ZnO	83 and 36	-	Antioxidant and antibacterial	[69]
*Jatropha curcas*	Crude latex	FeO	20–42	300–800	Wastewater treatment	[51]
*Juniperus procera*	Leaves	Ag	23	424	Antimicrobial	[70]
*Lonicera japonica*	Flowers	Au	10–40	530–580	Anticancer	[71]
*Lythrum salicaria*	Leaves	Ag	20–138	395–415	Antimicrobial, anticancer, and catalytic degradation	[72]
*Melia azedarach*	Leaves	Ag	14–20	420	Antibacterial, wound healing, antidiabetic, and antioxidant	[73]
*Mentha pulegium*	Leaves	ZnO	40	370	Antibacterial	[74]
*Mimusops elengi*	Fruits	Ag	43	431	Antibiofilm, antibacterial, and anticancer	[75]
*Moringa oleifera*	Seeds	Ag	17.6	421	Wound dressing	[30]
*Moringa oleifera*	Peel	GdO	26 ± 2	280–300	Antifungal, nontoxic, and photocatalyst	[29]
*Moringa Oleifera*	Leaves	FeO	18–20	668	Drug delivery	[76]
*Myristica fragrans*	Fruits	ZnO	66	200–700	Antibacterial	[77]
*Nymphaea alba*	Root	Au	32–280	200 and 300	Antibacterial and anticancer	[78]
*Ocimum Americanum*	-	ZnO	21	316	Antioxidant and antibacterial	[79]
*Ocimum basilicum*	Leaves	ZnO	10–25	370	Antibacterial	[80]
*Ougeinia oojeinensis*	Leaves	Ag	5–100	450–500	Antioxidant and antimicrobial	[81]
*P. austroarabica*	Leaves	Ag	16.8 ± 5.4	-	Catalytic efficacy and antioxidant	[82]
*Panax ginseng*	Roots	Zn	59.76 nm	340	-	[83]
*Phyllanthus acidus*	Leaves	ZnO	27.14–35.7	375	Anticancer and antioxidant	[84]
*Picrasma quassioides*	Leaves	Ag	5–40 nm	412	Radio sensitizing	[85]
*Pimpinella anisum*	Seeds	Ag-Au	16–48 and 15	428 and 544	Antioxidant and antimicrobial	[86]
*Pithecellobium dulce*	Peel	ZnO	30	250–300	Antifungal and photocatalytic activity	[87]
*Polyalthia longifolia*	-	Ag	45	443	Antiamoebic	[88]
*Prosopis juliflora*	Leaves	Ag	10–20	420	Wound healing and degradation	[89]
*Psidium guajava*	Leaves	Ag	5.88	425	Antibacterial and antioxidant	[90]
*Raphanus sativa*	Roots	ZnO	15–25	372	Wound dressing for diabetic foot ulcers	[91]
*Rhodiola rosea*	Rhizome	Ag	10	437	Antioxidant and catalytic reduction	[92,93]
*Rhus javanica*	Bark	Ag	67	400–435	Antioxidant, antibacterial, and catalytic	[46]
*Ricinus communis*	Seeds	ZnO	10–30	-	Antioxidant, antifungal, and anticancer	[94]
*Rubia cordifolia*	Leaves and roots	ZnO	257.1 ± 0.76	285	Antimicrobial and antioxidant	[95]
*Rumex hastatus*	Bark	Ag	61	400–435	Antioxidant, antibacterial, and catalytic	[46]
*Sauropus androgynus*	Leaves	ZnO	12–23	373	Antineoplastic agent	[96]
*Scoparia dulcis*	Leaves	Ag	3–18	420	Antimicrobial	[97]
*Scutellaria barbata*	-	Au	400–1000	525	Anticancer	[98]
*Senna alata*	Leaves	Ag	25	434	Antibacterial	[99]
*Senna auriculata*	Flowers	FeO	160–300	300 and 310	Antibacterial	[100]
*Sida acuta*	Leaves	ZnO	32.82	373	Antioxidant and photocatalytic activity	[101]
*Silybum marianum*	Seeds	Ag	13.20	448	Antioxidants	[102]
*Sonchus asper*	Leaves	TiO_2_	9–22	-	Antimicrobial	[103]
*Tabernaemontana heyneana*	Leaves, stem, and callus	ZnO	6.69	370–376	Antioxidant, anti-inflammatory, antidiabetic, anticancer, and photocatalytic activities	[104]
*Terminalia mantaly*	Leaves	Ag	11–83	350–700	Antibacterial	[105]
*Triphala churna*	-	FeO	29–74	-	Anticancer and super paramagnetism	[106]
*Vaccinium Arctostaphylos*	Leaves	ZnO	12.4 and 21	365	Antidiabetic, antibacterial, and oxidative	[107]
*Vallarai Chooranam*	-	Ag	43.1	432	Antibacterials, antioxidant, larvicidal, anti-acetylcholinesterase, and anticancer	[108]
*Withania coagulans*	Leaves	Ag	14	200–700	Antimicrobial	[109]
*Withania coagulans*	Berries	FeO	16 ± 2 and 18 ± 2	249	Antimicrobial	[110]
*Zea mays*	Corn flour powder	Ag	-	420	Antioxidant	[111]
*Zingiber officinale*	Rhizome	Ag	18.9–23.8	438–443	Antibacterial	[112]

## 3. Microbe-Mediated Synthesis of Metallic Nanoparticles

Biological entities secrete primary and secondary metabolites that serve as reducing agents to produce NPs from metal salts. Identification of such metabolites helps to manipulate the resultant NPs, often with the desired shape and size [113]. For instance, the size and distribution of bio-PdNPs were controlled by adjusting the ratio of microbial biomass and palladium precursors in a study reported by Zhang et al. (2022) [114]. The results revealed that the high bacterial cells (*Shewanella oneidensis)*-to-Pd ratio had the smallest average particle size of 6.33 ± 1.69 nm. Moreover, these bacteriogenic PdNPs with small size and uniformly distributed achieved a completely catalytic reduction of 200 mg/L Cr (VI) solution within 10 min, while the commercial Pd needs at least 45 min to do the same catalytic activity.

There is a significant variation in the ability of microorganisms to transform diverse types of metal ions into nanoparticles. This is dictated by several factors related to microbial physiology and intrinsic metal properties. Microbes have developed specialized detoxification mechanisms for certain metals they commonly encounter, allowing them to reduce remarkably high concentrations of those ions. Additionally, the redox activity of biomolecules secreted by microbes for reduction and stabilization purposes varies in its ability to reduce metal ions [115]. It is also reported that the binding affinity of metal ions to intracellular reductive proteins and metabolites also impacts transformation efficiency [116]. Beyond optimal levels, excess free metal ions exert toxic effects through oxidative stress, limiting the concentration range and permitting microbial transformation activity [117]. These physiological adaptations and redox characteristics governing uptake, reduction, and binding interactions determine the metal-specific resistance and transformation capabilities observed among microorganisms. In the respective studies, Rafeeq et al. (2021) demonstrated the remarkable capability of the fungus *Pleurotus floridanus* to convert a substantial concentration of 300 mM zinc ions into ZnNPs [118]. Similarly, Manimaran et al. (2022) reported that the fungus *Hypsizygus ulmarius* exhibited the ability to synthesize ZnNPs using a metal precursor of 5 mM [119]. Furthermore, Gharieb et al. (2023) conducted a study focusing on the biosynthesis of SeNPs, where they identified two endophytic fungal isolates, namely *Penicillium citrinum* and *Rhizopus arrhizus*. These fungal strains demonstrated remarkable tolerance up to 40 mM Na_2_SeO_3_ and achieved a remarkable conversion rate of over 99.0% when transforming 3.0 mM selenite into SeNPs [120].

**Figure 2:**
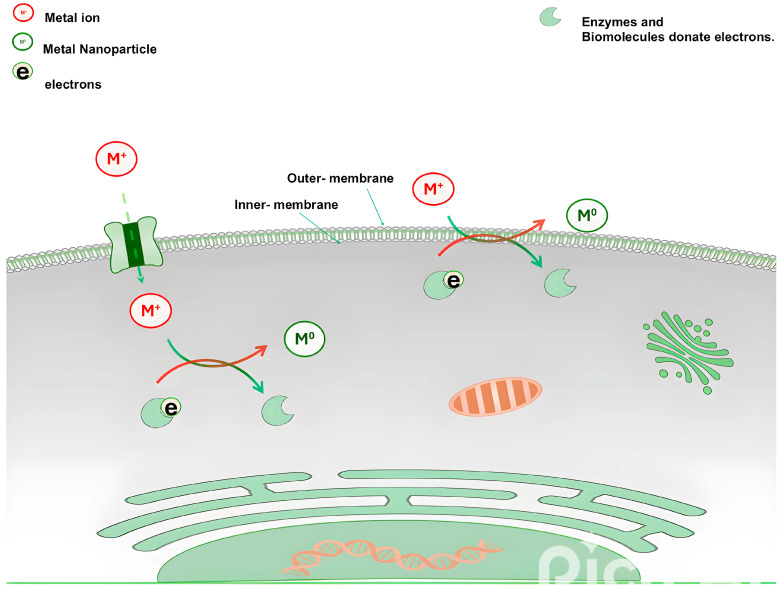
Suggested mechanism of metallic nanoparticle biosynthesis by microbes intracellularly and extracellularly. Intracellular synthesis occurs inside the cell, while extracellular synthesis occurs out the cell. Red and green arrows represent the oxidation-reduction reactions that carried out and result in the formation of nanoparticles.

When microbial cells are exposed to metals, their protective response may facilitate the unintentional formation of metal nanoparticles through bioreduction of ions by intracellular proteins, enzymes, cofactors, and metabolites, thus immobilizing the metals in less toxic forms [9]. On the other hand, non-specific binding to cell walls by microbes can potentially facilitate the reduction in metal cations [121,122]. Thus, microorganisms in general hold the innate potential to modify metals, resulting in the formation of metallic nanoparticles at the cellular level during their natural response to metal stress. Figure 2 illustrates the suggested mechanism of metallic nanoparticle biosynthesis intracellularly and extracellularly. Table 2 illustrates a huge assortment of metallic nanoparticles biosynthesized by microbes, including algae, bacteria, and fungi.

Currently, biogenic nanoparticles are believed to be superior to their synthetic analogs, as the first is more environmentally friendly, less costly, and easily available. In this respect and to test this notion, Silva et al. (2022) [123] studied the biosynthesized AgNPs mediated by *Aspergillus niger* ecotoxicity on some organisms, namely, *Daphnia similis*, *Danio rerio* (zebrafish), and *Chlorella vulgaris,* where the findings detected some side effects, highlighting the importance of avoiding any environmental adverse consequences when developing nanoparticles in the future.

**Table 2:** Microbe-mediated biosynthesis of metallic NPs.

Organism	Mode of Synthesis	NPs	NPs Size	UV Abs.	Ref.
**Fungi**					
*Agaricus bisporus*	Extracellular	Cu-NPs	2–10 nm	551 nm	[124]
*Aspergillus terreus*	Extracellular	Ag-Cu NPs	20–30 nm	517 nm	[125]
*A. Niger*	Extracellular	AgNPs	10–100 nm	430 nm	[126]
* A. austroafricanus *	Extracellular	AgNPs	2–51.34 nm	400 nm	[127]
*Penicillium oxalicum*	Extracellular	AgNPs	60–80 nm	--	[128]
*P. oxalicum*	Extracellular	CdO-NPs	40–80 nm	250–650 nm	[129]
*P. duclauxii*	Extracellular	AgNPs	3–32 nm	300–900 nm	[130]
*P. oxalicum*	Intracellular	CdO-NPs	22.94 nm	297 nm	[131]
*Metarhizium robertsii*	Extracellular	CuNPs	15.67–62.56 nm	670 nm	[132]
*Cordyceps militaris*	Extracellular	ZnO-NPs	1.83 nm	354 nm	[133]
*Enoki mushroom*	Extracellular	AgNPs	10 nm	435 nm	[134]
* Flammulina velutipes *	Extracellular	AgNPs	21.4 nm	----	[135]
* Ganoderma applanatum *	Extracellular	AgNPs	58.77 nm	435 nm	[136]
*Xylaria acuta*	Extracellular	ZnO-NPs	34–55 nm	280–500 nm	[137]
* Pleurotus florida * * (oyster mushroom) *	Extracellular	Au-Pt NPs	Au 17.96 nm Pt 23.45 nm	521 nm	[138]
*P. sajor-caju*	Extracellular	AuNPs AgNPs	Au 15–20 nm Ag 16–18 nm	Au 426 nm Ag 531 nm	[139]
*Ganoderma lucidum* *(reishi mushroom)*	Extracellular	AgNPs	15–22 nm	400–460 nm	[140]
*A. sydowii*	Extracellular	AgNPs	1 and 24 nm	420 nm	[141]
*Talaromyces purpureogenus*	Extracellular	AgNPs	30–60 nm	380–470 nm	[142]
*P. djamor*	Extracellular	TiO_2_-NPs	31 nm	345 nm	[143]
*Streptomyces* sp.	Extracellular	ZnO-NPs	12–35 nm	350, 400 nm	[144]
*Cordyceps militaris*	Extracellular	ZnO-NPs	10.15 nm	350 nm	[145]
*Alternaria* sp.	Extracellular	AgNPs	3 and 10 nm.	435 nm	[146]
**Bacteria**					
*Bacillus megaterium*	Extracellular	SeNPs	45.9 nm	200–900 nm	[147]
*Proteus vulgaris*	Extracellular	Iron oxide-NPs	19.23–30.51 nm	310 nm	[148]
*Vibrio alginolyticus*	Extracellular	AuNps	100–150 nm.	300–600 nm	[149]
* Lactobacillus * sp. *(LCM5)*	Extracellular	AgNPs	3–35 nm	420 nm	[150]
*Enterococcus* sp. *(RMAA)*	Intracellular	AuNPs	5–10 nm	360–660 nm	[151]
*B. cereus (SZT1)*	Extracellular	AgNPs	18–39 nm	418.99 nm	[152]
*Pseudomonas poae*	Extracellular	AgNPs	19.8–44.9 nm	422 nm	[153]
*B. siamensis*	Extracellular	AgNPs	25–50 nm	200–800 nm	[154]
*Cuprividus* sp.	Extracellular	AgNPs	10–50 nm	420 nm	[155]
*B. cereus RNT6*	Extracellular	ZnONPs	21–35 nm	250–800 nm	[156]
*Pseudomonas aeruginosa*	Extracellular	ZnONPs	6–21 nm	200–600 nm.	[157]
*Alkalibacillus* sp. *W7*	Extracellular	ZnONPs	1–30 nm	310 nm	[158]
*Pseudomonas putida (MCC 2989)*	Extracellular	ZnONPs	25–45 nm	200–800 nm	[159]
* Paraclostridium benzoelyticum *	Extracellular	ZnONPs	50 nm	300–800 nm	[160]
*P. haeundaensis*	Extracellular	AuNPs	20.93 ± 3.46 nm	535 nm	[161]
**Algae**					
*Cladophora glomerata*	Extracellular	ZnONPs	14.39–37.85 nm	290–360 nm	[162]
*Kappaphycus alvarezii*	Extracellular	ZnONPs	>100 nm	300–700 nm	[163]
*Ulva lactuca*	Extracellular	ZnONPs	12–17 nm	310 nm	[164]

### 3.1. Fungi

Bacteria, yeast, fungi, algae, plants, and even viruses became popular candidates in the field of metallic nanoparticle biosynthesis. Fungi, as one of these biological entities, have been tested for NP synthesis and were proven successful in producing targeted shapes, sizes, and functions. Moreover, fungal biological impacts are appreciated, as are their high metal tolerance and bioavailability [165].

Fungi show great promise as producers of metallic nanoparticles through biosynthesis owing to their diverse structures and metabolic abilities. As a kingdom, fungi include both multicellular organisms with thread-like mycelia for substrate colonization as well as unicellular yeast forms [166]. This mycelial nature allows extensive substrate penetration and interaction compared to other microbes. Additionally, the metabolic diversity between yeasts and filamentous fungi reflects their adaptation to different ecological niches [167,168]. As a result of their varied morphologies and biochemistry, fungi secrete an assortment of reducing and capping agents such as proteins, enzymes, and small molecules when exposed to metal salts [9]. These extracellular biomolecules facilitate the reduction in metal ions and stabilization of resultant nanoparticles [169], as shown in Figure 1. Fungi are thus advantageous green synthesis candidates due to their versatile cell architectures for metal uptake and active secretions governing bioreduction and shaping of nanoparticle properties. Their inexpensive cultivation and manipulation further promote the feasibility of fungal biosynthesis of nanomaterials.

Biosynthesis of NPs by fungi, also known as mycosynthesis, includes the harnessing of fugal biomass, cell-free extract, or cell-free filtrate to achieve the reduction of metal ions into nanoparticles. Akther et al. (2020) [170] used *Setosphaeria rostrata* endophytic fungus in the synthesis of silver nanoparticles. Similarly, Kumar et al. (2022) [171] synthesized zinc oxide nanoparticles (ZnO-NPs) applying *Aspergillus* sp. isolated extracellular products. Rafeeq et al. (2021) [118] documented the ability of *Pleurotus floridanus* culture filtrate to mediate the zinc oxide nanoparticle mycosynthesis. Equally interesting, *Colletotrichum siamense* extract in combination with tea tree oil was utilized as a base material in the making of silver-based nanoemulsions [172].

### 3.2. Algae

Algae has proven effective in metal ion reduction and thus the successful synthesis of metallic NPs. Algae can be cultivated under laboratory conditions as well as in large-scale production but also exist abundantly in nature. Algar has been used for the clean and green synthesis of metallic nanomaterials at a low cost [173]. Despite the success of algae as natural reducing agents, their role in the removal of toxic pollutants has not been fully discovered yet [174].

Unlike fungi and bacteria, algae-mediated biosynthesis of nanoparticles appears to rely heavily on the algal extract, which drives both reduction and stabilization of metal ions. In the study published by Yugay et al. (2020), polysaccharide extracts alginate, fucoidan, and laminaran were isolated from marine algae *Saccharina cichorioides* and *Fucus evanescens,* and their activity as a reducing and stabilizing agent in the biogenic synthesis of silver nanoparticles was evaluated [175]. Likewise, silver nanoparticles were synthesized by using an extract of *Spirogyra hyalina* as a capping and reducing agent [176]. Another study reported the green synthesis of stable silver nanoparticles (Ag-NPs) with an average size of 2.23–14.68 nm and copper oxide nanoparticles (CuO-NPs) with an average size of 3.75–12.4 nm using an aqueous solution of *Spirulina platensis* (blue green algae) as a reducing and capping agent [177]. Additionally, the algal extract of *Coelastrella terrestris* has been used for the biosynthesis of copper oxide nanoparticles (CuO-NPs) with enhanced photocatalytic and antibacterial properties [178]. Furthermore, platinum nanoparticles (PtNPs) were synthesized using an aqueous extract of the red algae *Halymenia dilatata* and exhibited characteristic biomedical applications [179].

Lichens offer a novel source for the green synthesis of nanoparticles as they represent a symbiotic partnership between fungi and algae, or cyanobacteria. As symbionts, lichens contain a diverse array of phytochemicals and biomolecules sourced from both their fungal and photobiont partners [180]. While plants, bacteria, fungi, and algae have been widely explored for metal nanoparticle production, lichens remain an underexploited option in nanotechnology. In this context, silver nanoparticles (AgNPs) were successfully biosynthesized using an extract of the *Heteroderimia leucomela* lichen [181]. This study also reported that the GC–MS analysis of lichen extract revealed the specific lichen-derived reducing and capping agents responsible for the reduction of silver ions and stabilization of the resulting colloidal silver nanoparticles. Thus, harnessing lichen extracts presents a novel avenue for developing green nanomanufacturing methods utilizing the diverse biochemical repertoire of these symbiotic organisms.

## 4. Natural Extract-Mediated Biosynthesis of Metallic NPs

### 4.1. Flavonoid

Polyphenol compounds, or flavonoids, occurred naturally in several plants, exhibiting enormous biochemical activities. They are known for their enrichment in hydroxyl and carbonyl groups; thus, their spontaneous interaction with metal ions results in complexes with unique properties. For instance, they have antimicrobial, antiproliferative, and antioxidant abilities and can be colored and eventually fluorescent [182].

Quasi-spherical PbO (27 nm) nanoparticles biosynthesized from the aqueous extract of the plant powder (*Sageretia thea*) have shown a high flavonoid content of quercetin, myricetrin, kaempferol, as well as syringic acid, daucosterol, and triterpene. Taraxerol has been reported to be responsible for the reduction of lead acetate to lead oxide nanoparticles [183].

Eight flavonoids, namely, taxifolin, isosilychristin, silydianin, silychristin, isosilybin B, isosilybin A, silybin A, and silybin B, are the main components of silymarin. Silymarin reduced the gold ions, forming silymarin-coated gold nanoparticles following the protocol described earlier by Kabir et al. Silymarin proved effective as a hepatoprotective and antifibrogenic agent [184].

### 4.2. Terpenoids

Terpenoid are associated with the biosynthesis of metal nanoparticles. For example, the formation of AuNPs from the reaction of HAuCl_4_ 3H_2_O with *Euphorbia peplus* leaf extract. The FTIR analysis of extract revealed the presence of cycloartenol, peplusol, 24-methylenecycloartanol, and lanosterol involved in the reduction of Au^3+^ ions to Au^0^ [185]. Similarly, the biosynthesized iron nanoparticles were formed by the reduction of FeCl_3_·6H_2_O using aqueous *Ageratum conyzoides* extracts (roots, stalks, and leaves) in an in vitro model. Some secondary metabolites from these extracts were identified using GC–MS and displayed a high content of terpenoids as cadala-1(10)3,8-triene, sesquiterpene alcohol (caryophyllenyl alcohol, cubenol, and globulol), triterpene (a-amyrin, friedelan-3-one), and rotundene [186]. 

### 4.3. Proteins

**Figure 3:**
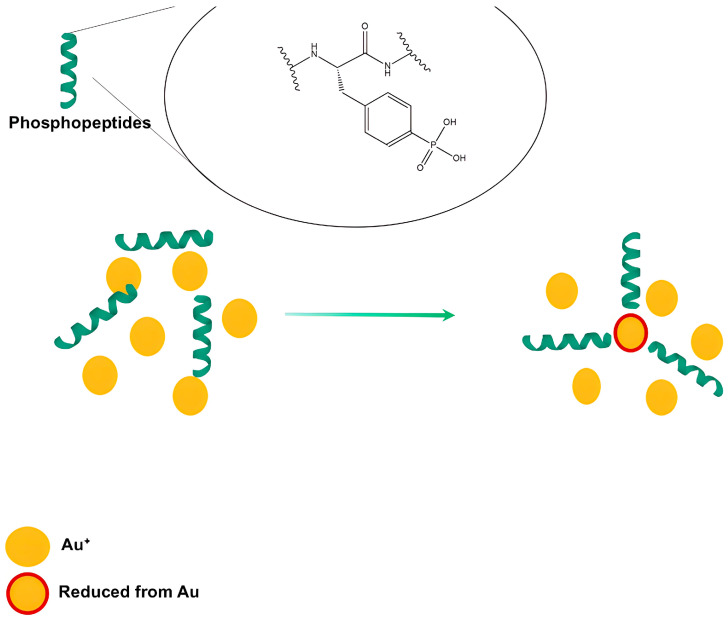
Phosphotyrosine peptide-stabilizing AuNPs [188].

The involvement of proteins in reducing and stabilizing metal nanoparticles was demonstrated [187]. FTIR analysis of ZnO-NPs produced from biomass filtrate of *Pseudomonas aeruginosa* showed different groups such as C=O, O–H, NH, and SH thiol groups preventing NP agglomeration. Moreover, the FTIR spectrum declares –NH stretching vibration and N–C=O amide bond, which is a typical phenomenon of the occurrence of proteins among the biosynthesized Ag-NPs from *Aspergillus fumigatus*. In addition, O–H stretching in flavonoids, alcohols, and phenolic compounds was observed [188]. AuNPs stability was indicated by the presence of the phosphate group as shown in Figure 3; the peptides bind to AuNPs surface through the thiol group [188]. Peptides without phosphate groups cannot stabilize the AuNPs; hence, tyrosine, serine, and threonine are the common amino acids that can undergo phosphorylation. Table 3 provide various natural extract that had been implicated in the biosynthesis of metallic NPs.

## 5. Factors Affecting the Biosynthesis of Metallic NPs

There are several factors that affect the biosynthesis of metal nanoparticles; some factors affect the rate of biosynthesis, like light. Another feature is the morphology of the NPs, as they may be spherical, platelets, dodecahedra, and rods by using photo-induced reaction protocols. The size of the particle plays a critical role in determining if this particle is nano or not, as size can affect the utilization of this NPS. Finally, pH can alternate the mechanism of synthesis. These different factors, including reaction time, pH, temperature, metal precursor, and biomass concentration, significantly influence the biosynthesis of metallic nanoparticles using various sources of plant extract, fungi, algae, or bacteria [198,199]. Ansari et al. (2023) reported the maximum production of silver nanoparticles by Neem (*Azadirachta indica*) was noted at 70 °C after 3 h of reaction time following the addition of 10 mL of 1 mM silver nitrate [200]. Another study reported by Gharieb et al. (2023) revealed that pH, temperature, and concentration of metal salt markedly influenced SeNPs production by *Penicillium citrinum* and *Rhizopus arrhizus* [120]. In this section, we will discuss each of these factors.

### 5.1. Light

Most or all plants use light to make photosynthesis, which results in the formation of ATP, NADPH, and NADH. NADH plays a major role in the synthesis and electron transfer to metal ions and converting them into metal zero-valence atoms. Therefore, light has a main role in NPS synthesis. Sunlight reduces the required time for NPS biosynthesis as it influences the electron transfer to metal ions. The wavelength of light has an effect on the size and morphology, so it provides a control button in synthesis [201].

### 5.2. Temperature and Heating Rate

Any natural occurring reaction can be affected by heat and temperature, so temperature is considered a controller. The higher the temperature, the faster the NPS synthesis and the more uniform the produced nanoparticles are. The formation of NPS has two important steps affected by the temperature. Firstly, nucleation, which is influenced by high temperatures, leads to the formation of small NPS. Nucleation as a chemical process includes the reaction between starting materials and reducing agents, as well as the electron transfer chain. Secondly, crystal growth is influenced by low temperatures, resulting in large NPS. Crystal growth is a physical process that depends on adsorption and balance between charges. Also, it is a thermodynamics process, so it is affected by temperature [202].

### 5.3. pH

The pH controls the stability of NPS; for example, nanoparticles are stable at the isoelectric point, as the coagulation or flocculation as well as the double-layer alteration is influenced notably by the change in pH [203]. Furthermore, spherical and larger nanoparticles were seen with higher pH, supporting the notion that pH directly influences the size and shape of NPs [204].

### 5.4. Time

When we speak about the rate of NPS biosynthesis, we speak about the amount of reducing metal ions to zero-valent metal atoms per unit of time, so when the synthesis occurs in a short time, the NPS that is formed is small and vice versa [204].

### 5.5. Reactant Concentration

When we speak about reactant concentration, we speak about the starting material concentration. All plants and microorganisms have suitable concentrations for the biosynthesis of NPS. However, it is different from plants and microorganisms; it is also different from the species in the same genera. But in general, in plants, if the initial concentration is high, it causes aggregation for formed NPS, which increases the size of NPS. In microorganisms’ high concentration leads to inhibition of growth, as in high concentration it becomes toxic [205].

### 5.6. Reducing Agent Concentration

Chemical reactions that occur inside the living cell depend on some factor; one of them is the reactant. Synthesis of NPS inside a living cell: the reaction happens between starting materials that are present and obtained from the environment to the inside of the plant and the reducing agent that is used to reduce and convert the metal ions into a metal zero-valence atom and convert it to NPS. As any reaction occurs in nature, it needs sufficient initial concentration to start the reaction, and when the concentration of the reducing agent is high, it reacts with the starting material that is present in the reaction field faster, which makes the entering of starting materials into the reaction faster, so the synthesis of NPS occurs at a high rate, and the size of NPS is also affected [205].

### 5.7. Capping Agents

A capping agent consists of polar and non-polar groups impacting its functionality, where the head is representing the polar side, responsible for the interaction with the metal atom, and the hydrocarbon tail is the non-polar side responding to the surrounding medium [206]. Capping amphiphilic molecules is hence considered a stabilizer, controlling the nanoparticle size by preventing aggregation and stopping overgrowth within the medium of preparation [207]. The stability of metal nanoparticles is an important factor to determine the effectiveness and suitability for use in different fields. Instability can lead to aggregation, loss of bioactivity, or alterations in physicochemical properties. Capping agents play an important role in the stability of these nanoparticles from different biological systems, offering a green and eco-friendly alternative to traditional chemical methods [122]. Clarance et al. (2020) reported the stable synthesis of gold nanoparticles aided by camptothecin produced by Fusarium solani associated with Chonemorpha fragrans [208]. Moreover, silver nanoparticles were successfully produced from the leaf extract of *Achillea millefolium*. The synthesized nanoparticles show long-term stability due to the presence of flavonoids and phenols such as tannic acid [31]. Similarly, Majeed et al. (2021) reported the extracellular synthesis of iron oxide nanoparticles by Proteus vulgaris. The functional groups such as hydroxyl alkane and amines are responsible for the stabilization and formation of FeONPs; also, the stability of the nanoparticles was evaluated through zeta potential analysis measured at 79.5 mV [165].

### 5.8. Pressure

NPS shape and size are influenced greatly by the applied pressure; for instance, at ambient pressure conditions, a faster reduction reaction where metal ions are transformed into zero-valence metal atoms was observed [209].

## 6. Encapsulation of Metallic Nanoparticles

**Figure 4:**
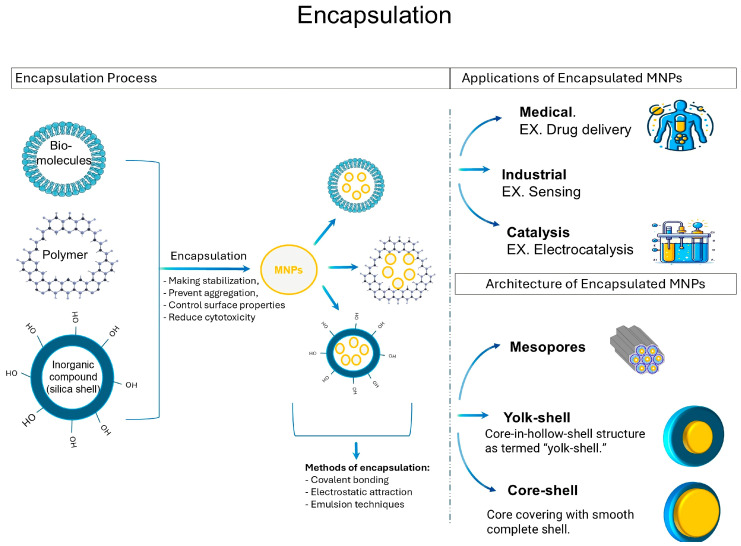
Illustration of the process of encapsulating MNPs with various polymers and techniques and gives insights into its applications and architecture.

Metallic nanoparticles are encapsulated through a process of functionalization that involves applying a protective shell along their exterior surfaces. A key goal of encapsulation is to stabilize nanoparticles by using coating materials to form barriers surrounding the core structures [210]. The shell serves to persist in the interface between the nanoparticle and its surrounding environment. This improves critical properties such as colloidal stability, biocompatibility, ability to enable controlled release of compounds, and reduction in toxic effects [211,212,213]. A variety of encapsulated materials can be utilized to coat the nanoparticle exterior completely or partially, including polymeric membranes, inorganic casings, and lipid/protein-based formulations, as shown in Figure 4.

Polymeric shells are often used as they can be easily applied and provide biocompatibility. Polymers like chitosan, PEG, and PVP are electrostatically bound or crosslinked to form a flexible nanoshell around the nanoparticles [214,215,216]. Inorganic shells like silica provide rigidity through techniques like sol–gel processes. They enhance stability and can endure harsh environments [217]. Inorganic nanostructures like titania and zirconia can also encapsulate metallic nanoparticle cores [218]. Lipid shells such as phospholipid vesicles are suitable for biomedical uses as they mimic cell membranes. Protein coats from albumin or collagen are biodegradable and non-toxic. These organic shells allow the conjugation of ligands and controlled release [219,220]. The choice of encapsulating material depends on the desired application. The primary methods for encapsulating metallic nanoparticles include covalent binding, layer-by-layer assembly, self-assembly, emulsion techniques, sol–gel processes, crosslinking, and electrostatic deposition [221]. Covalent binding uses functional groups on the coating to chemically attach to the nanoparticle surface. Layer-by-layer assembly forms multilayered coatings through alternating layers of oppositely charged materials deposited electrostatically. Self-assembly relies on hydrophobic/hydrophilic interactions to form coatings like lipid bilayers spontaneously in solution [221]. Emulsion techniques disperse nanoparticles in an organic phase before water emulsification enables polymer deposition. Sol–gel processes grow inorganic shells like silica through hydrolysis and condensation reactions [222]. Crosslinking strengthens coatings through chemical or photonic linking. Electrostatic deposition uses charge attractions between nanoparticles and coatings from solution [223]. The method choice depends on factors like nature of coating, stability requirements, and type of surface functionalization needed.

Encapsulation shields the core material while also enabling surface modification for diverse applications involving drug delivery, catalysis, and sensing. The systemic toxicity of drugs is decreased by the effective loading of drug molecules inside nanocarriers. Selenium nanoparticles HA-Se@DOX were tested against human cervical carcinoma (HeLa cells), and the results demonstrated both inhibition of the proliferation and promotion of the apoptosis in vivo [224]. Similarly, copper oxide nanoparticles synthetized by *Pterocladia capillacea* red algae and loaded with nedaplatin exhibited excellent anticancer activity against ovarian cancer, hepatocellular carcinoma, and breast cancer cell lines [225]. Taken together, green synthetized nanoparticles loaded with cytotoxic drugs have a superior impact on cancer cell lines in comparison with the free drugs, with an obvious targeted function and apoptosis induction in vitro [226].

## 7. Applications of Green Synthesized MNPs

**Figure 5:**
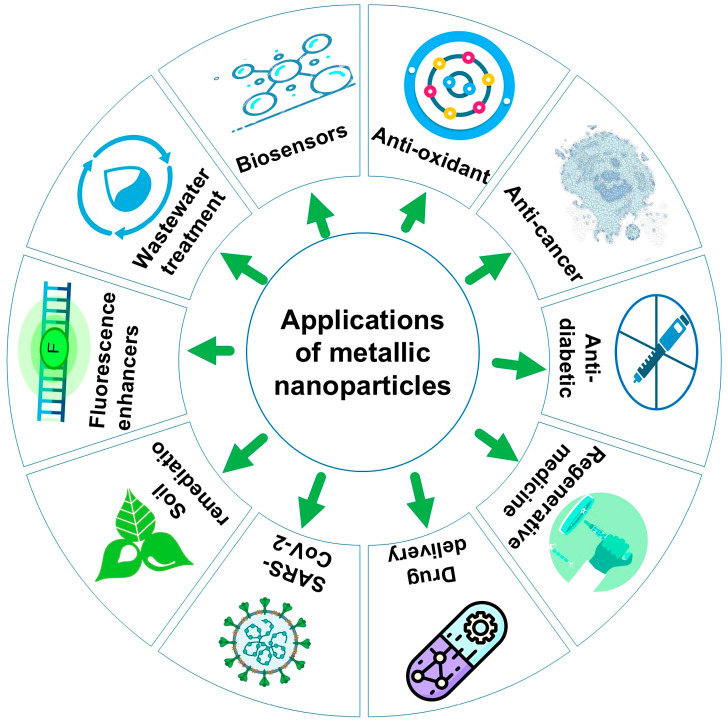
Applications of green synthesized metallic nanoparticles.

Green-synthesized NPs are widely used in several applications. For that reason, it has a variety of biomedical, environmental, and other industrial implications, as shown in Figure 5. In recent years, most of these applications have been oriented toward medical applications, as stated in Table 1, where 85 out of 90 applications focused on the medical field. These applications include drug delivery, wound healing, anti-microbial, anti-biofilm, anti-cancer, and anti-amoebic activities that were detected in many plant species. *Abutilon indicum, Achillea millefolium,* and *Achillea wilhelmsii* were among those involved in the synthesis of MnONPs, AgNPs, and AuNPs, respectively [27,30,31].

Recent publications in the medical field indicate that green synthesized nanoparticles are promising for regenerative medicine and tissue engineering. For example, *Raphanus sativa* ZnONPS was used in wound dressing for diabetic foot ulcers [91]. Moreover, *Allium saralicum* AuNPs promote wound healing; hence, they work as antioxidant molecules to prevent inflammation on the site of injury [227]. On the other hand, *Jatropha curcas* and *Cinnamomum tamala* FeONPs were used with wastewater treatment (bacterial portion), dye adsorption, and toxic metal removal [50].

### 7.1. Medical Applications of Metallic NPs

#### 7.1.1. Antioxidant Properties

MNPs act as perfect scavenging agents for reactive oxygen species (ROS). MNPs possess surface properties that enable them to interact with ROS and convert them into less harmful species, thereby reducing oxidative stress [228]. This process of reducing the oxidative reaction occurs directly by neutralizing ROS or indirectly by chelating the metal ions from transition metals [229]. For example, there is evidence that AuNPs can prevent DNA damage and apoptotic cell death caused by H_2_O_2_ [230]. 

#### 7.1.2. Anticancer Properties

The MNPs gain much importance in the medical field due to their ability to cause many disruptions inside the cancer cells, such as DNA damage, misfolded protein, mitochondrial dysfunction, and enzyme deactivation leading to cell death [231,232]. Due to the acidic PH in the cancer cell, which leads to the release of Ag^+^, these free Ag^+^ ions promote the generation of ROS, damaging the mitochondrial DNA and causing cell death [232].

Breast cancer is the second-leading cancer diagnosed globally. Every year, about two million new incidents are reported. HER2/neu oncogene overexpression causes an unfavorable prognosis for most breast cancer patients. In a recent study, the inhibitory effect of gold nanoparticles synthesized using *Curcuma wenyujin* extract (CW-AuNPs) was examined against the expression of HER2/neu in breast cancer cell lines, and it was concluded that CW-AuNPs have an anti-cancer effect. In this regard, this study shows that CW-AuNPs promote the formation of ROS inside the breast cancer cell, which will inhibit the expression of HER2/neu, leading to growth suppression of cancer cells [57].

In another study, cancer cells were exposed to low concentrations of superparamagnetic iron oxide nanoparticles, which caused magneto-mechanical cell death [233]. This means MNPs that contain a paramagnetic element, such as iron nanoparticles, show promising cytotoxic activity against cancer cells when exposing the region of application to a magnetic field.

#### 7.1.3. Anti-Diabetic Properties

Diabetes is one of the most fatal diseases, causing around 1.5 million deaths each year globally, according to the World Health Organization. To be able to control diabetes mellitus, some chemical drugs such as acarbose and voglibose were used to suppress the action of α-amylase and α-glucosidase, which are essential enzymes for the conversion of carbohydrates into a simpler form, decreasing sugar concentration in the blood [234]. Most recently, they used zinc oxide (ZnO-NPs) as a potential eco-friendly alternative to chemical drugs, as they can inhibit α-amylase and α-glucosidase activities [235]. Moreover, needle-shaped AgNPs synthesized using *Linumisitatissimum* extract exhibit an antidiabetic activity with maximum inhibition for α-amylase and α-glucosidase by 79.84% and 58.86%, respectively, at 100 μg/mL [236]. In another study, cubic-shaped AgNPs synthesized using *Colpomenia sinuosa* showed significant inhibition for *α-*amylase and *α-*glucosidase activities with 90.50 ± 0.10 and 94.30 ± 0.10, respectively, at a concentration of 1000 μg/mL [237].

#### 7.1.4. Regenerative Medicine

MNPs have various applications in the fields of regenerative medicine and tissue engineering due to their small size, characteristic shape, mechanical strength, and most importantly, hemostatic ability [238]. Several studies show that constructing scaffolds from biologically active materials can promote their regenerative ability by two main mechanisms. The first is to enable cell-scaffold interaction, and the second is to release factors that aid in the regeneration process [239]. One of the studies shows the ability of AgNPs to support the process of bone tissue repair. There, the ability of pure polyether ether ketone (PEEK) was compared with PEEK that was carried on AgNPs. It was found that AgNPs could promote cell proliferation and increase alkaline phosphatase activity. Furthermore, AgNPs play a significant role as an antibacterial agent against both Gram-positive and Gram-negative bacteria [240].

In another study, selenium nanoparticles derived from *Proteus mirabilis* YC801 promoted nerve regeneration and stabilized the microenvironment. For instance, SeNPs remarkably increase the number of neurons, preserve the integrity of the spinal cord, and promote the production of M2-type macrophages, which are the immune cells of the brain; therefore, inflammation is suppressed. Moreover, SeNPs reversed the spinal cord injury SCI-mediated production of reactive oxygen species [241]. Also, nanocomposites synthesized by one of the green methods were applied for the treatment of Alzheimer’s disease (AD) [242]. Because of the cubic Qu@P-80@AuPd biocompatibility and high blood–brain barrier (BBB) permeability, it has a huge capacity to remove intracellular amyloid-β (Aβ) and hinder the neurotoxicity induced by Aβ. Aβ is a protein responsible for high neurotoxicity as it damages the cerebral cortex, releasing neurotoxins leading to AD [243]. Additionally, Nanoparticles (NPs) synthesized by honeybee products serve dual purposes in preventive and interceptive treatment strategies due to their richness in essential metabolites [244].

#### 7.1.5. Drug Delivery

The application of MNPs in drug delivery is driven by three key rationales: their targeted ability, controlled releases of required doses, and their ability to enhance drug stability and bioavailability in the body [245]. In terms of targeted drug delivery, MNPs can be coated with specific molecules that bind only with specific cell surface receptors and act as carriers to the exact site [246]. Moreover, MNPs act as smart carriers, which deliver medications directly to their targets, protect them from degradation while traveling in the patient’s body, whether in the circulation or the digestive system, and release them in controlled amounts.

In a recent study, results indicated that the chitosan (CS-MnFe_2_O_4_) NP with pH-sensitive properties makes an interesting candidate for intestinal-targeted drug delivery through oral administration by preventing drug release in highly acidic gastric fluid [247].

#### 7.1.6. SARS-CoV-2

In a recent study, the ability of small-sized MNPs to participate in the fight against COVID-19 was proven. Metallic nanoparticles have been explored for their potential against COVID-19 in the field of pharmaceutical nanotechnology. They can exhibit direct antiviral activity by interfering with viral entry and replication. Moreover, metallic nanoparticles can serve as drug delivery systems, enhancing drug stability and targeted delivery of antiviral drugs. They have been utilized in the development of diagnostic tools, providing enhanced sensitivity and specificity in detecting SARS-CoV-2. Metallic nanoparticles can also be used for surface coatings, reducing viral transmission by inactivating the virus on various surfaces. [248,249].

### 7.2. Environmental Applications

Along with the environmental crisis, nanotechnology can participate in solving many urgent problems, such as wastewater and pollutant degradation [250].

#### 7.2.1. Biosensors

Wastewater pollution can be explained by the contamination with the pesticides and fungicides used in agriculture, mostly of unstable compounds. These compounds easily degrade to give more toxic compounds in the environmental water, which lately causes many health problems. In this case, green-based MNPs were used to detect these hazardous compounds in an accurate way. AgNPs were introduced in a surface plasmon resonance (SPR) device to act as the sensing elements that interact with the analyte molecules. The changes in the SPR signal resulting from the binding or adsorption of analytes onto the MNPs are then detected and utilized for detection, offering high sensitivity and resolution in various applications [251].

Additionally, MNPs are considered a label-free detection method. This means that it does not require the use of molecular labels or tags such as fluorescence for the analyte of interest [252]. For example, AgNPs are used to detect the presence of many compounds (the analyte) resulting from the decomposition of Mancozeb (a worldwide fungicide used on a large scale in agriculture), as the decomposition produces ethylene thiourea (ETU) and ethylene bis iso thiocyanate (EBIS) and other minor degradation products such as glycine and ethylene urea (EU) [253]. 

#### 7.2.2. Wastewater Treatment and Catalytic Reduction

Green-synthesized nanoparticles are used for water and wastewater treatment, as they are effective in removing pollutants and catalyzing degradation reactions [254].

Many health problems can be caused by toxic compounds such as 4-nitrophenol and 4-aminophenol. Exposure to these substances can result in skin irritation, eye disorders, and respiratory problems. For this reason, Ag-NPs showed excellent catalytic activity in reducing 4-nitrophenol to 4-aminophenol and then to a lesser toxic form [255,256]. Moreover, the green synthesis of gold nanoparticles (NPs) using grape pomace waste has shown efficiency in recycling and removing heavy metals and organic pollutants from wastewater [257].

#### 7.2.3. Soil Remediation

Nanomaterials aid in carrying fertilizers and pesticides. Controlling the release of nutrients and protecting the crops from pests [258].

## 8. Conclusions

In conclusion, the field of metal nanoparticle synthesis through green chemistry using natural extracts has witnessed significant advancements and achievements during the period from 2019 to 2023. The biosynthesis of metallic nanoparticles (MNPs) mediated by plants, microbes (fungi, algae, and bacteria), and natural extracts has emerged as a promising and environmentally friendly approach. It offers several advantages, such as cost-effectiveness, scalability, and the utilization of sustainable resources. Through the review, we have explored the biosynthesis mechanisms involved in these green synthesis methods, highlighting the role of various factors such as pH, temperature, precursor concentration, capping agents, light, and agitation. The encapsulation of MNPs within natural polymers has also been investigated as an effective means to enhance stability, controlled release, and targeted delivery of these nanoparticles. Additionally, the diverse applications of metallic nanoparticles in different sectors have been discussed, showcasing their potential in fields such as medicine, catalysis, electronics, and environmental remediation.

## 9. Future Prospective

Moving forward, the field of metal nanoparticle synthesis through green chemistry using natural extracts holds immense potential for further advancements and applications. The exploration of novel natural sources, including diverse plant extracts, microbial strains, and other natural resources, presents an exciting avenue for discovering new bioactive compounds capable of efficient MNP synthesis. By deepening our understanding of the underlying biosynthesis mechanisms, such as enzyme-mediated reactions and gene expression, we can gain greater control over the synthesis process and optimize parameters for enhanced MNP production. Additionally, efforts should focus on tailoring nanoparticle properties such as size, shape, composition, and surface modifications to optimize their performance in specific applications. Comprehensive studies on the biocompatibility and potential toxicity of green-synthesized MNPs will pave the way for safe and responsible utilization in various fields. Bridging the gap between laboratory-scale synthesis and large-scale production is crucial, and scalable and economically viable methods should be developed to facilitate industrial implementation. By addressing these future perspectives, we can unlock the full potential of green-synthesized MNPs, propelling their practical applications across sectors and contributing to sustainable technological advancements.

## Figures and Tables

**Table 3:** Metabolites used in the synthesis of metallic NPs.

Plant Species	Metabolites Identified	Class	Metal NPs	Ref.
Prickly pear *Opuntia* (peel extract)	-------------	Proteins and carbohydrates	Se	[189]
*Hibiscus sabdariffa*	Kaempferol, Quercetin Cryptochlorogenic acid, neocholorogenic acid, caffeoyl shikimic acid	Flavonoid Acid	Au	[190]
* Fenugreek * seed extract	--------------------	Saponin, proteins, and polyphenols	Ag-Fe_3_O_4_	[191]
(*Cuminum cyminum*) Cumin oil	Quinoid	Phenolic	Ag	[192]
*Nigella sativa* seed extract		Steroids, tannins, flavonoids, coumarins, cardiac glycosides, saponins, and diterpenes	Ag	[193]
*Salvia officinalis* and *Thymus serpyllum* ethanolic and hydroalcoholic extracts	Gallic acid Protocatechuic acid Caftaric acid Chlorogenic acid Caffeic acid *trans p*-coumaric acid *trans* ferulic acid Rosmarinic acid Rrutin hydrate Chlorophyll a and b	Acid Flavonoid chlorophyll	Mesopores of silica and titania nanomaterials	[194]
*Terminalia mantaly* ™ extracts of leaf, root and stem/bark		Alkaloids, phenolic content, flavonoids, tannins, triterpenes, glucosides, saponins, anthraquinones, and steroids	Au	[195]
*Euphorbia peplus* ethanolic leaf extract (EpExt)	Obtusifoliol Cycloartenol Peplusol 24-methylenecycloartanol, lanosterol, 24-methylenelanosterol, and 9-cis-tricosene Angelic acid	Steroids Triterpene acid	Au	[185]
*Leucas aspera* leaf extract	----------------------------	Polyphenols and proteins	Ag	[196]
*Xylocarpus granatum mangrove* (bark extracts) and *Avicennia officinalis* (leaf extract)	--------------	Phenolic compounds	Ag	[197]
